# A review of the ESL/EFL learners’ gains from online peer feedback on English writing

**DOI:** 10.3389/fpsyg.2022.1035803

**Published:** 2022-10-26

**Authors:** Siyi Cao, Siruo Zhou, Yong Luo, Tao Wang, Tongquan Zhou, Yizhong Xu

**Affiliations:** ^1^School of Foreign Languages, Southeast University, Nanjing, China; ^2^School of Foreign Studies, Nanjing University of Posts and Telecommunications, Nanjing, China; ^3^School of Foreign Languages, Beijing Institute of Technology, Zhuhai, China; ^4^School of Psychology, Qufu Normal University, Qufu, China; ^5^College of Foreign Languages, Nanjing University of Aeronautics and Astronautics, Nanjing, China

**Keywords:** online peer feedback, EFL/ESL writing, gains, theories, educational psychology

## Abstract

Peer feedback is essential in writing English as a Second/Foreign Language (ESL/EFL). Traditionally, offline PF was more widely favored but nowadays online peer feedback (OPF) has become frequent in ESL/EFL learners’ daily writing. This study is undertaken to probe into the gains of using OPF in ESL/EFL writing on the basis of 37 research articles published in core journals from 2012 till 2022. In order to accurately cover the previous researches, we capitalize on three methods to evaluate and analyze the data, i.e., database search, citation search and manual search. Results show that from the perspective of the ESL/EFL learners’ gains, the OPF is basically divided into two categories (cognitive OPF and affective OPF), involving eight aspects in all: face-based strategies, revision-based comments, writing performance, learning environment, reflection/critical thinking/responsibility, writing emotion, motivation, and attitudes; and OPF can be well supported by a set of theories like Process-oriented Writing Theory, Collaborative Learning Theory, Interactionist Theory of L2 Acquisition and Vygotsky’s sociocultural theory. By comparison, the gains from OPF outperform those from offline PF in many dimensions (e.g., face-based strategies), despite some overlaps (e.g., the shift of the role) that were revealed in several investigations. Based on the past studies, we propose some pedagogical implications on OPF from ESL/EFL writing, including accenting the “student-centered” teaching strategy, providing students with OPF on the basis of incremental knowledge, adopting OPF regularly in ESL/EFL writing activities to shape personalities and outlooks and putting OPF into its full play with recourse to abundant internet-based vehicles. This review is desired to provide a guideline for both the peer feedback practice and the upcoming scholarly researches with respect to EFL/ESL writing.

## Introduction

Peer Feedback (also known as peer review, peer response, peer editing, peer evaluation or peer revision) refers to the activities in which students work together to provide comments on their own written or oral drafts by active communications in an academic subject (Liu and Edwards, 2018). Since 1970s, peer feedback has been widely used in L1 writing classes to encourage students to evaluate their peer’s drafts and solve diversified issues *via* text modifications (Bruffee, 1984; Gere, 1987; Spear, 1988). Gradually, this type of modernized pedagogy has also been intensively introduced as an instructional means in ESL/EFL (English as a second/foreign language) writing classes since 1990 (Henfernik, 1983; Bell, 1991; Carson and Nelson, 1996; Min, 2006; Chen, 2016). A review of previous studies shows that peer feedback brings students a set of benefits or gains in many ways. Specifically, peer feedback enables students to experience and enhance collaborative writing (Nunan, 1993) and increases learners’ autonomy. Besides, it also fosters the sense of multiple readers and raises writer’s awareness (Stanley, 1992; Zhu, 1995; Berg, 1999; Min, 2005; Chen, 2016).

Peer feedback in ESL/EFL writing is subdivided into two types, offline and online types in terms of modality (Peeters, 2018; Ahmed and Abdu, 2021). Offline peer feedback refers to the traditional modes like face-to-face peer feedback (FFPF). FFPF allows students placed into groups to assess and evaluate their peers’ drafts, requiring them to provide comments in a face-to-face classroom (Mendonca and Johnson, 1994; De Guerrero and Villamil, 2000; Chen, 2016; Saeed et al., 2018). Previous researches related to FFPF in ESL/EFL writing focused on many aspects, such as language functions of feedback patterns (e.g., exploratory function), potential benefits (e.g., improve linguistic details) and factors affecting FFPF (e.g., instructions prior to FFPF) (Stanley, 1992; Mendonca and Johnson, 1994; Zhu, 1995; Min, 2005; Liou and Peng, 2009; Hanjani and Li, 2014; Saeed et al., 2018).

Online peer feedback (OPF) arises from the development of electronic media around the end of 20*^th^* century. These media, which are mainly network-based or web-based discussion boards, made OPF prevail in ESL/EFL writing classrooms since 1990 (Braine, 2001), mounting to its peak in 2020 because of the COVID-19 pandemic (Rimmer, 2020). Against the coronavirus background, online courses became the primary means of delivering instruction for all classes (Alsuwaida, 2022); hence, computer-mediated peer feedback in place of the traditional mode served as the dominant tool in L2 writing classes. However, the debate has been open about whether OPF works better than the traditional peer feedback in EFL/ESL writing. For instance, Song and Usaha (2009) and Pham (2020) found the OPF-group students showed more revision-oriented comments and global revisions than the FFPF-group students. According to Ebadi and Rahimi (2017), OPF using Google Docs outperformed FFPF in four aspects of academic writing skills (i.e., task achievement, coherence and cohesion, lexicon and grammatical range and accuracy). Some other studies show that traditional FFPF is superior to OPF. In the study by Guardado and Shi (2007), ESL students did not address a higher percentage of important comments in OPF because they felt unconfident and quite shy in negotiating and clarifying the meanings with peers. Liu and Sadler (2003) discovered that students in FFPF group made more subsequent revisions. However, OPF group produced more revision-oriented comments. Given that the writing scores were highly improved in FFPF group than in the networked group, Braine (2001) accented the cautious use of technology for peer feedback in writing classes.

Online peer feedback (OPF) and FFPF are often adopted combinatorically so as to compare their effects on L2 English writing class practice. Warschauer (1996) made a comparison on the equality of students’ participation either in FFPF discussion or in OPF discussion. The author found that the students showed more equal participation in computer mode, for they felt comfortable using more complex sentences in OPF. In DiGiovanni and Nagaswami (2001), students put more focus on tasks in electronic mode when engaging in combined modes of OPF and FFPF. In particular, teachers participated more in learners’ communication and helped them discuss in an appropriate direction during this process. Chang (2012) demonstrated that the incorporation of OPF and FFPF was favorable for peer response, but individuals differed in mode preference. Tai et al. (2015) combined the teacher-led feedback and OPF, revealing that students in combination group improved a lot in writing skills (e.g., content, organization and grammar). Accordingly, the authors suggested that more OPF discussions should be encouraged than the FFPF discussions to give students equal opportunities to express themselves.

Although mixed findings concerning the effects of OPF have been found in early studies, the gains of using OPF in ESL/EFL writing account for the absolutely largest proportion. For instance, Chen (2016) concluded the characteristics, pros and cons of OPF on the basis of 20 articles from 1990 to 2010. Saeed et al. (2018) reviewed 37 articles and categorized different patterns (i.e., language functions, factors affecting OPF and FFPF) of learners’ interactional feedback exchanges into two classes, FFPF and OPF. Based on 17 primary studies, Lv et al. (2021) conducted a meta-analysis of the effectiveness of different online feedback, including peer, teacher and automated feedback, revealing that the peers’ online feedback had larger effect size (*g* = 0.777) than the online automated feedback (*g* = 0.696).

The studies mentioned above indeed proved OPF had a lot of gains, but left the following issues unsettled: (1) although numerous studies in the past decade have shown the benefits of OPF from ESL/EFL writing, their majority just focus on special aspects (e.g., language functions), ignoring other gains students may obtain from writing peer feedback; (2) although there have been a few studies on OPF from 2012 till 2022, all of them (including reviewing articles) focused on other aspects instead of gains of OPF from ESL/EFL writing; (3) although the positive impacts of OPF are multi-faceted yet heterogeneous in nature, there has not been a systematic classification of the gains to date; (4) although a couple of theories were introduced to account for the availability of the gains from ESL/EFL writing, their explanatory power was not compared and hence may not be sufficiently convincible, particularly from the perspective of educational psychology. As a consequence, the current study aims to describe and generalize the gains of using OPF in ESL/EFL writing from different perspectives (e.g., face-based strategies) by exhaustively reviewing all the research articles published in the core journals from 2012 till 2022. In addition to solving the above issues, this study highlights our contribution mainly from two aspects, the wider coverage of literature concerned and the more aspects regarding the gains of OPF.

Specifically, this paper is dedicated to answering the following three questions:

(1) How many types of gains from OPF in ESL/EFL writing can be identified in the researches from 2012 to 2022?

(2) What theories can be adopted to account for the different gains from the OPF in ESF/EFL settings?

(3) What implications can be acquired from the current review, from the perspective of educational psychology in particular?

## Methods

The present study used three methods to review the literature systematically, including database search, citation search and manual search. In order to target at the articles for review accurately, we adopted the following inclusion and exclusion criteria throughout the searching process.

Inclusion criteria:
(1) The articles were related to ESL/EFL writing activities and published between 2012 and 2022.
(2) The articles were targeted to study at least one type of peer feedback (i.e., OPF) in ESL/EFL writing activities.
(3) All articles applied qualitative or quantitative methods or mixed methods.

Exclusion criteria:
(1) The articles were published before 2012 and their topics were not related to OPF, such as special needs, corrective feedback etc.
(2) The OPF studies selected the participants from ESL/EFL learners other than English natives.
(3) The articles used anonymous participants to explore OPF from English writing activities.

For database search, the authors first employed the databases of ERIC, SCOPUS, Web of Science and CNKI to explore all the articles related to the benefits of OPF from 2012 to 2022. Only peer-reviewed studies were selected as the target articles for analysis by the keywords *OPF, second language learning, foreign language learning* and *writing contexts.* Papers dealing with special needs, corrective feedback, self-correction and automated feedback were excluded from the search. As a result, 74 records were identified, i.e., 23 articles from ERIC, 6 articles from CNKI, 22 articles from SCOPUS and 23 articles form Web of Science database. After removing 37 duplicated articles, the database search led to a total of 37 peer-reviewed papers pooled for analysis.

At the screening period were adopted citation search and manual search. For citation search, re-read the 37 papers *via* database search to discover more related articles by their references. For manual search, use Google scholar to search for other papers of the same topic. In all, citation and manual search led to another 6 relevant papers. Taken together, a total of 43 full-text articles were selected and assessed for eligibility.

**FIGURE 1:**
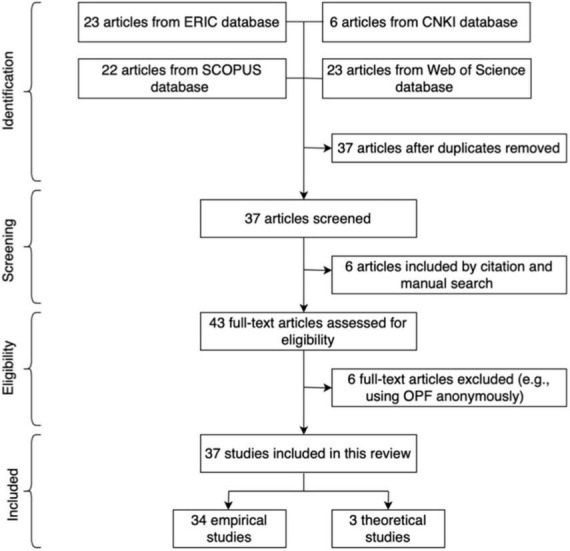
Screening process of the reviewed papers.

To guarantee the research focus of studies, 6 articles irrelevant to the benefits of OPF (e.g., those using OPF anonymously) were excluded after a re-examination and a primitive analysis. Eventually, 37 articles (i.e., 34 empirical and 3 theoretical studies) were selected as the most relevant ones to look into the gains from OPF (the other detailed information like education background is listed in Appendix 1). The screening process of the reviewed articles was shown in Figure 1. In order to display a systematic overview of the OPF, all the articles were sorted out in an excel file according to feature maps (Hart, 2001) in terms of title, author/date, research questions, methods, materials, results, abstract and given a final category for further classification.

## Discussion

According to the results obtained from the above methods, this section discusses two issues relating to the gains from a variety of OPF in EFL/ESL writing. One describes how these potential gains are realized in EFL/ESL writing activities, and the other introduces theories approaching OPF from other perspectives, particularly the educational psychology.

### A panorama of the gains from the online peer feedback on ESL/EFL writing

**FIGURE 2:**
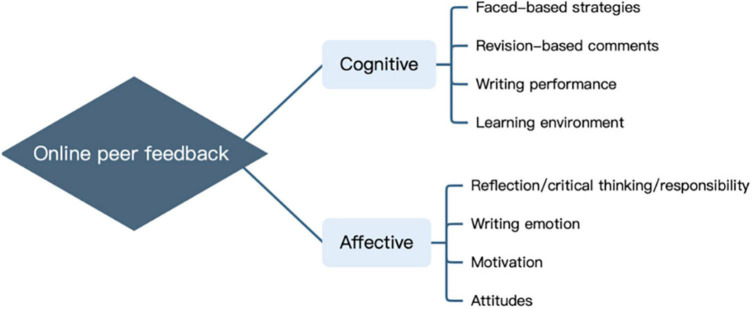
Classification of online peer feedback (OPF).

Based on the 37 articles selected, the beneficial patterns of OPF in EFL/ESL writing contexts are roughly categorized into two classes, cognitive OPF and affective OPF. Cognition deals with mental processes such as memory, learning, problem-solving, attention and decision making, employing concrete and manageable strategies while affect deals with emotional areas, such as motivation, attitudes, and feelings (Sfard and Kieran, 2001; Jones and Issroff, 2005; Immordino-Yang and Damasio, 2007). In terms of this distinction, cognitive OPF and affective OPF can be further divided into four sub-aspects, respectively (as shown in Figure 2): cognitive OPF involving face-based strategies, revision-based comments, writing performance and learning environment; and affective OPF involving reflection/critical thinking/responsibility, writing emotion, motivation, and attitudes. Here, critical thinking is classified into affective OPF due to its involvement of willingness, desire, and disposition to base one’s actions and beliefs (p. 23) (Siegel, 1989). Reflection captures the conceptualization of knowledge, thoughts and feelings of students, which were used to detect affective outcomes (YuekMing and Abd Manaf, 2014). According to the affective experiences described, the students deemed that they had the responsibility to participate in learning apart from their perceptions of learning itself (Galloway et al., 2016). The three aspects are discussed altogether just because they have some shared points, like desire, motivation, belief, and feeling among other internalized emotions. The eight types of OPF in turn yield the corresponding eight gains, specifically. The following is to elaborate on all the aspects to illustrate how the OPF gains are realized in ESL/EFL writing activities, with the gains from cognitive OPF followed by the gains from affective OPF.

#### Gains from cognitive online peer feedback

##### Gains from face-based strategies

Among the 37 articles, 4 papers are involved in the gains from face-based strategies, demonstrating that the students in OPF could overcome face embarrassment, and provide or receive praise and critique from their peers (Daweli, 2018; Saeed et al., 2018; Ma, 2020; Pham et al., 2020).

For example, Pham et al. (2020) explored the perception of students from the impacts of Confucian values by virtue of OPF, showing that the students’ negative feelings at the beginning shifted significantly to a positive level after OPF. This indicates that learners broke through the face-based cultural barriers by resorting to OPF. Such a change of face-based strategy was also found in Ma (2020) that the students were willing to give more praises than suggestions (685 vs. 394) and tried to be less face-threatening when providing online comments.

When interviewed about whether or not preferring to receive comments from peers *via* Google Docs, the participants responded that “Your best friend’s comments are preferred because he is closer to you than the teacher.” (Daweli, 2018: p8), suggesting that using Google Docs-mediated peer feedback can aid all participants to avoid face embarrassment, for hierarchical power and students’ beliefs and experiences of peer feedback were supposed to be the key to this face culture (Ma, 2020). Based on the literature review of OPF’s language functions, Saeed et al. (2018) concluded that students gave more social interactional comments, such as thanking peers, praising peers and surprise, to adjust the face-based strategy in order to maintain good relationships with peers, which were not yet found in offline peer feedback like FFPF.

With regard to face culture, OPF evidently outdoes FFPF from their gains from English writing, for the students engaged in FFPF tend to strongly avoid assessing and commenting on their peers’ drafts due to face threatening (Chiu, 2009), particularly in Confucian contexts (Pham et al., 2020). Confucian values involve two core principles, the concept of face and power distance. It is reported that the students in the Confucian context evade providing comments and giving critiques to their peers, for they fear destroying the harmonious relationship and causing conflict or even hurting their classmates (Chiu, 2009). Similarly, Chinese students are reluctant to voice criticism and express disagreements due to face culture (Luo and Liu, 2017). That is, they more like and respect teacher feedback which is endowed with reliable knowledge and absolute authority (Li et al., 2010).

##### Gains from revision-based comments

Two papers in the current review showed that OPF could generate more revision-based comments than FFPF in actual writing practice (Ho, 2012; Pham, 2020). According to Pham (2020), OPF using Google Doc was better than traditional oral FFPF in this regard. The results suggested that more revision-oriented comments were triggered by the OPF group (MO = 1.14) than by the FFPF group (MF = 0.53) for Vietnamese EFL students. Nevertheless, there occasionally came up a bit different situation in which both OPF and FFPF produced a similar proportion of revision-oriented comments (53% vs. 52%) (Ho, 2012).

Another 6 articles explored revision-oriented comments from OPF in two types, i.e., global and local comments. Our statistics showed that the two types of comments do not come up symmetrically in students’ writing peer feedback. While some studies indicate that more local comments (e.g., referencing, supporting details and language) were made than global comments (e.g., content, organization and argumentative genre) in OPF (e.g., Chang, 2012; Saeed et al., 2018), others prove the opposite view (e.g., Bradley, 2014; Pham and Usaha, 2016; Saeed and Ghazali, 2017; Ma, 2020). To illustrate, Chang (2012) reported that 87% of local comments but 13% of global comments appeared in OPF. By contrast, Ma (2020) found that peer suggestions were more about layout and organization (125) than about language details (43).

##### Gains from writing performance

A critical concern in ESL/EFL writing OPF is whether students can make evident progress on writing performance under OPF. In our reviewed literature, there are 7 papers investigating this issue centered on the students’ improvement in the writing scores. For instance, Usaha (2020) discovered that 32 Vietnamese EFL students’ writing scores were significantly enhanced after OPF. Similarly, Kitchakarn (2013) and Huang H. Y. C. (2016) reported that the writing scores were elevated significantly after blog-mediated peer feedback and Wechat-mediated peer feedback, respectively, indicating that OPF played an important role in improving students’ writing skill.

Other studies focus on comparing OPF with other types of feedback in terms of writing scores. For instance, Ciftci and Kocoglu (2012) compared the writing scores of two modes, i.e., blog-mediated peer feedback and FFPF. They found that both groups improved their writing scores after peer feedback but the average writing scores were significantly higher from blog-mediated feedback (*M* = 69.19) than from FFPF (*M* = 65.17). The same circumstance exists in Wahyudin (2018) and Awada and Diab (2021) that the writing scores improved significantly in the OPF group than in the FFPF group. Likewise, the comparison among the writing scores of three groups (i.e., self-correction, paper-pencil peer feedback, and electronic peer feedback) reveals that although all the types of feedback could increase learners’ writing achievements, the electronic peer feedback group performed the best (*M* = 73.74) among the three (Wanchid, 2013).

In addition to the writing scores, the 7 papers have shown that after OPF, both local and global aspects of writing were all improved. For example, OPF is shown to improve error correction (i.e., grammatical errors, spelling errors and sentence correction) and text revisions (e.g., global features of text), particularly for less-proficient students (Yang and Meng, 2013). Pham et al. (2020) also obtained a similar result from students’ perspective in the Confusion context in Vietnam. In Pham et al.’s (2020) questionnaire survey to test students’ perceptions toward global and local aspects of writing, global aspects were more favored by the students, as “E-peer feedback will help (helped) to improve the flow, organization, and transitions of the essays.” Besides, students focused more on local aspects like grammar, sentence structure, and vocabulary after OPF (Li, 2012; Saeed and Ghazali, 2016).

Relative to other types of peer feedback (e.g., paper-based peer feedback, FFPF and automated corrective feedback), OPF also facilitates the improvement of local and global areas of writing performance. OPF group made more local (i.e., word and sentence) and global (i.e., substitution, reordering and consolidation) revisions whereas paper-based peer feedback group made no such revisions (Yang, 2016). OPF using the Google Docs group can make significantly more writing enhancements than the FFPF group (Ebadi and Rahimi, 2017). In Shang’s (2022) study of local features by OPF and automated corrective feedback, the grammatical errors by OPF (*via* Moodle) were proven to be significantly fewer (M = 10.85) than the errors by automated corrective feedback (*via* the Coll Sentence Corrective Network) (*M* = 13.79). For lexical density, the students adopting OPF produced more tokens (*M* = 245.50) and types of words (*M* = 132.91) than the students adopting automated corrective feedback. Similar findings were also unveiled in other studies (Cai, 2012), indicating that OPF offers students more gains than other types of feedback from evaluating writing performance.

##### Gains from learning environment

Of the 37 targeted articles, 5 studies displayed that OPF was easy to help build up a better learning environment and the corresponding gains were classified from two aspects, the use of the target language and the atmosphere during the feedback (Daweli, 2018; Saeed et al., 2018; Ma, 2020; Elboshi, 2021; Sun and Zhang, 2022).

With respect to the target language used on online platforms, students tend to apply English as the communicational language when providing comments orally on each other’s assignments on a web-based platform (where the teacher is monitoring, and the first language is not allowed) (Elboshi, 2021). In addition, L2 learners are required to write suggestions for their peers in English; hence, the OPF-targeted English practice forces them to write more and create a more active learning atmosphere during the class. Sun and Zhang’s (2022) translanguaging study showed that students using translanguaging (*M* = 12.15) through OPF outperformed those in English-only OPF (*M* = 11.23). Yet the students claimed that both conditions were conducive to improving learning efficiency, implying that either the use of English or translanguaging as the target language in OPF helps offer an academic learning environment and enhance L2 learners’ understanding.

Online peer feedback (OPF) generates a more open and friendly atmosphere, encouraging students to express more opinions freely. According to Saeed et al. (2018), compared with offline peer feedback, students added more social interactional comments (e.g., thanking, welcoming, praising and even social talks or chatting) to establish a more positive atmosphere in OPF. Similar evidence was also revealed by Ma (2020), in which the praises by students doubled peer suggestions (685 vs. 394) in a wiki writing assignment, suggesting that OPF situation relative to FFPF is more comfortable so that peers try to be more friendly and supportive in providing comments. Another study about OPF employing Google Docs suggests the gains from the comments students made, as exemplified by “I really like how it saves my time instead of meeting in class and work wherever I want.” (Daweli, 2018: p9). This indicates that the Google Docs-mediated peer feedback was just like working outside a classroom, creating for the peers a free social and open environment.

#### Gains from affective online peer feedback

##### Gains from reflection/critical thinking/responsibility

Reflective thinking, as the synonym of critical thinking emphasizes on how students express their thoughts and feelings about what has occurred when making decisions (Schön, 2017). More importantly, responsibility was also considered as an important trait for developing the habit of thinking critically (Djoub, 2021). That is why the three items are interrelated and therefore combined together for discussion here.

According to the 4 articles in the reviewed literature, OPF accelerates students’ reflection, critical thinking and responsibility to some extent. In Zhang et al.’s (2014) study, an interviewed student claimed that “…My peers’ feedback in blogs gives me an opportunity of knowing where I am wrong and why I am wrong. That encourages my self-reflection of my writing.” This suggests that OPF helps to facilitate critical self-reflection and learners can gain a rewarding experience of L2 writing through a self-reflection process. As mentioned above, Pham et al.’s (2020) investigation of Vietnamese learners’ attitudes toward the reflective and critical effects after OPF showed that OPF significantly improved their reflective thinking in correcting mistakes and minimizing weaknesses.

Critical thinking makes students take the initiative in taking responsibility for their learning in OPF. When interviewed about their feelings in the process of OPF using Google Docs, Saudi EFL students responded, “It is a positive feeling because I feel I am a critical editor” (Daweli, 2018: p8). Similar gains of OPF were supported by a study using Facebook (Wahyudin, 2018), suggesting that Facebook-based OPF on English writing made students more aware of errors or mistakes and improved students’ critical thinking in writing, resulting in the significant increase of their writing ability. According to Ma (2020), the critical comments from peers’ OPF could also predict the L2 writing scores of writing assignments by correlation analysis (r = –0.559). This implies that L2 learners likely enjoy the responsibility of providing OPF and improving their peers’ writing (Cassidy and Bailey, 2018).

##### Gains from writing emotion

In the present review, 2 articles dealt with writing emotions, demonstrating that OPF is a valid method to mitigate the effects of writing anxiety for L2 learners. With recourse to Second Language Writing Anxiety Instrument (SLWAI),^1^ Iksan and Halim (2018) compared the degree of writing anxiety for Malaysian L2 learners after OPF between OPF group (using wiki) and FFPF group. The results showed that both groups could low L2 writing anxiety and OPF was more effective than FFPF. Similarly, with 41 South Korean English majors, Bailey and Cassidy (2019) also adopted SLWAI to explore the same research question and found that OPF could reduce or eliminate writing anxiety.

As an important component of writing emotions, writing anxiety refers to the fear of the writing process that surpasses the possible benefits of the capacity to write (Thompson, 1980). In general, all writers (either native speakers or second-language learners) have experienced writing anxiety during the writing process (Cheng et al., 1999; Woodrow, 2011). The two articles justified the point that OPF provides an effective way to reduce or low writing anxiety for L2 learners. However, few studies dealt with this topic in the past, requiring more its investigations in the future.

##### Gains from motivation

Motivation is the process that initiates, guides, and maintains goal-directed behaviors and plays a very important role in ESL/EFL learners’ writing. The 4 articles of the reviewed literature demonstrate that students greatly enhance motivation *via* OPF when learning English writing.

Huang J. (2016) adopted a questionnaire to survey Taiwan students’ perceptions of learning motivation, such as “I feel writing blog assignments is easier and more motivating than doing other writing assignments.” The results showed that this item had the highest mean score in three classes (*M* = 4.62) because the assignment *via* OPF did not have defined topics, so students enjoyed more latitude to choose what they wanted to write about.

The other three studies (Cai, 2012; Zhang et al., 2014; Qiu and Li, 2022) relating to OPF motivation were all conducted in China. By interviewing students about why they were all motivated by OPF, Cai (2012) thought that the students tended to be afraid of losing face in the Confucian culture context; meanwhile, every student could gain praise from peers, bringing them a sense of achievement. Zhang et al. (2014) employed a 36 student-based questionnaire to study the relationship between blog-mediated peer feedback and learner motivation from two aspects: self-efficacy (e.g., “I can do the hardest work in my WRITING class if I try.”) and task value scale (e.g., “I find WRITING interesting”). Their result showed that OPF was correlated to learner motivation (*r* = 0.450) and three factors seemed to explain the motivation: (1) immediacy and availability of OPF; (2) attention from the intended readers; and (3) protecting one’s face, indicating that students increased confidence by using OPF.

Qiu and Li (2022) compared the motivational effects of OPF and teacher feedback on English writing. In their research, motivation was divided into two parts, intrinsic writing motivation and extrinsic writing motivation. The statistical analysis showed that higher scores in achievement motivation (a subtype of intrinsic writing motivation) occurred in OPF than in teacher feedback (MO = 3.986; MT = 3.681), suggesting that OPF was more effective than teacher feedback in terms of generating intrinsic writing motivation.

These results are in line with Aljumah (2012), which demonstrated that incorporating web tasks (e.g., blog) into writing courses could enhance ESL/EFL students’ writing motivation and effectiveness because they felt that all the people in the world (their teachers and peers in particular) were reading their writing. In this regard, OPF acts as an incorporating tasks using online platforms, indeed boosting L2 students’ writing motivation.

##### Gains from attitudes

A total of 10 articles (Ciftci and Kocoglu, 2012; quantitative study: Chen, 2012; Kitchakarn, 2013; Wanchid, 2013; Huang J., 2016; Cassidy and Bailey, 2018; Xu and Yu, 2018; Putra et al., 2021; qualitative study: Kitchakarn, 2013; Ebadi and Rahimi, 2017) have unanimously demonstrated that ESL/EFL students had positive attitudes toward the OPF practice.

In a survey to explore students’ perceptions of OPF, Ciftci and Kocoglu (2012) found that 86.7% of students strongly agreed upon the assistance of blogs-mediated peer feedback in improving their English and 80.01% would recommend to other students the online writing course using blogs. Additionally, 73.30% of students thought that the features of blog websites could help writers a lot. Similarly, in the study by Cassidy and Bailey (2018), 89% students believed that OPF helped them enhance their writing ability and 60% admitted that they made improvements according to their peers’ comments.

The questionnaire-based studies have acquired similar results. For instance, Chen (2012) and Wanchid (2013) designed a different number of statements to test students’ attitudes toward three types of feedback, including self-correction, paper-pencil peer feedback and electronic peer feedback, and the results pointed to the same conclusion that electronic peer feedback received the highest scores from most of the participants. Responses like “The peer feedback activity was a useful learning tool to improve my writing ability” (Kitchakarn, 2013) conveyed the similar gain obtained from the student’s experiences in using OPF. In the same fashion, Huang J. (2016) reported that the participating students adopted a supportive attitude toward the blog-mediated peer feedback. Putra et al. (2021) showed that 73% of the students (*M* = 3.77) were willing to provide peer feedback by Ozone.

Interview is another approach to examine ESL/EFL learners’ attitudes toward OPF. According to Kitchakarn (2013), 24 out of 34 participants reported that peer feedback *via* blogs was useful for learning from mistakes and gaining more vocabularies and writing skills. ESL/EFL students in the research (Ebadi and Rahimi, 2017) liked peer-editing using Google Docs because they could learn from peers by comparing the writings to put focus on the key features, such as core ideas in feedback.

Opposite to the above were the studies by Choi (2007) and Guardado and Shi (2007). In Choi (2007) Hong Kong L2 l earners reserved attitudes toward OPF while Canadian ESL/EFL students showed mixed attitudes in Guardado and Shi (2007). These diversified findings may result from the situation in which some students considered online communication more challenging than face-to-face communication, easily leading to misunderstanding in writing classrooms.

To summarize, among the 37 articles divided into the two categories, more studies dealt with cognitive OPF (31) and relatively few with affective OPF (20), despite some overlapping articles for different issues. This asymmetrical distribution may result from the easy access to cognitive OPF test in data collection, e.g., the data regarding whether the writing performance was improved during OPF. By contrast, the data relating to affective OPF are hard to obtain and lead to few studies as a consequence. For instance, the conclusion that OPF could enhance responsibility of ESL/EFL students was drawn solely from the interview (Cassidy and Bailey, 2018). In addition, 7 articles dealt with the comparison between OPF and FFTP, 5 articles between OPF and teacher feedback, automatic feedback or other types, further intensifying the important role of OPF in ESL/EFL writing activities.

### Major theories on the gains from online peer feedback

Ideally, specific theories had better be proposed to directly target at motivating why and how students can obtain gains from OPF on English writing. But according to the 37 reviewed articles, the theories adopted were basically lent from more general domains like educational psychology, social psychology and language acquisition. In practice, these theories do provide the rationales for the feedback and the corresponding gains related to ESL/EFL writing activities. In another way, students’ gains from the OPF can be well-explained by the hypotheses both theoretically and expirically. On this account, the following introduces 4 representative theories on the OPF gains, i.e., Process-oriented Writing Theory, Collaborative Learning Theory, Interactionist Theory of L2 Acquisition and Vygotsky’s sociocultural theory.

Process-oriented Writing Theory from educational psychology is considered a dynamic, non-linear and recursive process in which the writing takes place (Hayes, 2012). As one of the three steps constituting the process (Keh, 1990), the third step stresses that the text should be revised several times according to the feedback provided before completing the final assignment. In this regard, ESL/EFL students undoubtedly enhance their reflection/critical thinking/responsibility through some rounds of text revisions and improve their general writing ability by offering different feedback, including OPF.

Collaborative Learning Theory from social psychology argues that learning and knowledge can be constructed implicitly under social communications among peers, which can be regarded as a process wherein knowledge like language skills can be acquired through collaboration (Bruffee, 1984). In light of the theory, OPF provides a facilitative and learning environment so that the English learners are willing to offer revision-based comments wherein L2 learners can effectively complete the assignment (rather than do it individually), with the help of peers’ interaction and collaboration (Hu and Lam, 2010).

Interactionist Theory of L2 Acquisition from language acquisition stresses the important role of explicit and implicit feedback in second language learning, which creates opportunities for students to negotiate the meaning actively and discuss it with peers (Long and Porter, 1985). In line with this theory, students with recourse to online peer response offer adequate positive and negative feedback and then make modifications according to these inputs and bridge the gaps in their interlanguage system (Hyland and Hyland, 2006). Compared with the Collaborative Learning Theory, this theory appears to emphasize the mutual exchange of ideas when the participants serve as a text writer or a reviewer. The common point of the two theories is to highlight the mutual interaction or collaboration among language learners, in which peer feedback (the OPF in particular) fulfills the function directly, i.e., to improve writing ability *via* recurrent revisions and modifications of writing manuscripts.

The gains of reflection/critical thinking/responsibility of OPF draw support from Vygotsky’s sociocultural theory in the view of educational psychology, which accents the significance of social interaction among peers in learning and cognitive development (Vygotsky, 1987). In Vygotsky’s (1978: 86) view, students can develop from a novice level to a higher level with the assistance and scaffolding of an expert learner by improving reflection and critical thinking in the “zone of proximal development (ZPD)”.

Activity Theory, as the extension of Vygotsky’s theory, serves as another important theoretical framework for the gains of the learning environment, writing efficiency and overall writing quality of OPF in L2 writing (Jin and Zhu, 2010; Zhu and Mitchell, 2012; Yu and Lee, 2016). This theory, developed from the construct of mediation, holds the view that human beings mediate the relationships with others and the world through artifacts like physical tools (e.g., books and computers) and symbolic/psychological tools (e.g., language and signs) (Jin and Zhu, 2010). In this theory, mediated activities, which are socially organized and goal-directed, play an essential role in human development. Associated with ESL/EFL writing, OPF mediates students and the world through computers and offers them a more academic and friendly learning environment, facilitating collaborative learning among peers (Yu and Lee, 2016).

To conclude, among the current 37 reviewed articles, 17 papers considered Vygotsky’s sociocultural theory as their theoretical framework while 8 papers adopted Process-oriented writing theory to introduce OPF. Both Collaborative learning theory and Interactionist theory of L2 acquisition was used in 4 studies, respectively (but the left 8 articles were centered on the report of emperimental data, without resorting to any theory at all). This distribution may result from that Vygotsky’s sociocultural theory is one of the most significant theories in educational psychology. By contrast, Collaborative learning theory and Interactionist theory of L2 acquisition are from other fields.

## Comments and implications

### Positive aspects favoring online peer feedback studies from ESL/EFL writing

The current review combines the studies on online peer feedback from ESL/EFL writing in the past ten years, illustrating the feedback’s specific gains from students’ writing activities. As known to ESL/EFL learners, peer feedback acts as a helpful vehicle for improving their writing ability. The literature review shows that OPF overlaps with FFPF (face-to-face peer feedback) but differs in some aspects from the perspective of facilitating English writing.

To start with, both OPF and FFPF offer a chance for students to undergo a shift in role that they become more careful in providing revision comments to their peers (Ho, 2012). It is well acknowledged that teacher feedback as a component of teacher-centered activity tends to ignore the needs of students themselves, who act as a passive role in various class assignments (Bredo, 2012). By contrast, OPF and FFPF are student-dominated activities, in which the students temporarily experience the shift of role from “student” to “teacher” in the writing tasks and therefore become more reflective and responsible so that more revision-based comments are generated in these activities.

Next, the complementarity of different students’ English knowledge *via* OPF and FFPF improves their comprehensive writing ability. As claimed by Collaborative Learning Theory (Bruffee, 1984), English knowledge and meanings could be implicitly and explicitly acquired and constructed through social communications among peers because different people hold different perspectives according to their various cultural backgrounds and previous experiences. OPF and FFPF create a social learning environment where students can convey their knowledge, enrich their horizons through peers’ cooperation and then put their comprehensive thinking into their writing (Saeed et al., 2018).

In addition, peer feedback helps students to establish good habits, and a strict teacher’s eyes toward writing practice. Due to the shift from a “student” to a “teacher” in OPF and FFPF, students act in a more active role in making peer feedback, more attentive and more responsible (Cassidy and Bailey, 2018). As a result, they will become more critical of peers’ writing and offer as many suggestions and advice as they can for the peers.

Nevertheless, OPF is superior to FFPF in ESL/EFL writing practice. First, OPF assists in hindering face-to-face embarrassment so that peer feedback writing becomes more relaxed. It can be observed that students are reluctant to voice criticism and express disagreements because they fear destroying the harmonious relationship and causing conflict, even hurting their classmates, particularly in the Confucian context (Pham et al., 2020). OPF enables students to be involved in a more comfortable environment where they are willing and bold to face critique (Ma, 2020). For example, some modifications or mistakes can be pointed out directly by OPF.

Second, more time and more ready preparations are available for the participants when they are instructed to provide OPF instead of FFPF. As it is, FFPF requires instant assessments and comments by students, which is a great challenge, particularly for those who are intermediate or elementary in English proficiency (Yang and Meng, 2013). Opposite to FFPF, OPF does not compel students to give opinions about their peers’ writing drafts right now. Rather, it allows them to have enough time to think about how to produce appropriate comments on the papers to be evaluated. This is consistent with what has been discussed in the *Gains from Environment* above.

Third, OPF breaks through the space restriction between the writer(s) and the reviewer(s), therefore overcoming potential cultural barriers. More often not, spacial immediacy does not work (e.g., Cov-19 pandemic), and students are forced to stay far away from each other to listen to the class and participate in relevant learning activities (Rimmer, 2020). Under this situation, FFPF fails to come onto the stage, and OPF becomes the only way to fulfill learning exchange including ESL/EFL writing assessments. In addition to it, OPF offers another opportunity to co-participate in activities for people with various cultural backgrounds in remote places (Bada and Olusegun, 2015), breaking through the potential cultural barriers related to space restriction.

### Some aspects to be improved for future online peer feedback studies of ESL/EFL writing

Apart from the above positive achievements in past studies, this review reveals some aspects to be improved for further OPF explorations.

Firstly, most of the studies (i.e., 34 articles) were empirical. However, the fewest are theoretical (i.e., 3 articles), showing that researchers prefer to use statistical data to explore OPF issues (Saeed et al., 2018). At first glance, the data are very objective and reliable, for they are rigidly collected and measured. Nevertheless, what the figures mean/imply is reliant on creative thinking and philosophical speculation based on language acquisition and educational psychology. On this account, OPF’s theoretical studies ask for more space in both width and depth to explain the potential benefits or gains from the peer feedback of ESL/EFL writing activities.

Secondly, all the reviewed studies are uniformly based on students’ experience, i.e., what they do as OPF(e.g., in terms of questionnaires and interviews) (Pham, 2020). Little evidence was reported about the direction by teachers when students were making diversified feedback. Although OPF is a student-centered activity in ESL/EFL learning, teachers still play a fundamental role in guiding and monitoring students in the process of OPF (Zhan et al., 2022). However, no study of this kind has come up to date.

Thirdly, ESL/EFL learners’ OPF is affected by their culture they are in. Evidently, this is an important aspect in revealing the diversity and discrepancy of OPF between learners of different cultural backgrounds. Nevertheless, in the past ten years, only one study (i.e., Pham et al., 2020) has dealt with the topic. How is Asian culture (e.g., Confucian culture) different from European culture (e.g., Bible-based culture) as students provide feedback from ESL/EFL writing? How is non-English culture transferred into English culture? Such questions merit further exploration to reveal the similarity and diversity of OPF by ESL/EFL learners across cultures.

Fourthly, the reviewed literature is restricted in methodology. Specifically, all the studies used questionnaires and interviews to collect data (Ma, 2020). What if other methods (e.g., text analysis and OPF comparison) were adopted in OPF studies on ESL/EFL writing? More room is needed in this regard to increase the validity and reliability of OPF research.

Fifthly, some gains from OPF were mentioned by previous researches but not involved in this decade. However, these gains from OPF mentioned in our study are very common in the ESL/EFL writing activities. For example, the gains that OPF could bring more revision-based comments was also found by Liu and Sadler (2003) and Song and Usaha (2009). However, only two articles in this decade explored this topic.

### Pedagogical implications on online peer feedback on ESL/EFL writing

Online peer feedback (OPF) is not confined to English writing but functions as a regular practice in various pedagogical activities. What follows focuses on the potential implications of OPF from ESL/EFL writing.

First, the “student-centered” teaching strategy is to be more accented. As shown in this review, OPF is to have students enjoy more latitude in offering comments on the task(s) they are given. That is, OPF instantiates the student-centered pedagogical rationale, which contrasts with the traditional teacher-dominated pedagogy, easily leading to the depressive atmosphere in the classroom and the disharmonious tutor-student relationship. Accordingly, teachers should attempt to reduce the direct infusion of knowledge and offer students more freedom by shifting their role from good listeners to active participants in probing problems. In this regard, OPF is a good choice, the efficient strategy increasing their activity to learning, which as a pedagogical practice is supported by Choice theory (Glasser, 1998; Irvine, 2015), claiming that human has some basic needs to satisfy, such as freedom and fun.

Second, students are encouraged to provide OPF based on incremental knowledge. As known to all, acquiring knowledge is a step-by-step process, in which students can only give feedback on the given tasks in light of what they have already known. As a consequence, an English teacher should instruct the students to learn how to give comments on ESL/EFL writing according to their competence. As mentioned above, their feedback can start with local features (e.g., diction and grammar) and move to global features (e.g., discourse coherence and organization) as they progress in English proficiency and general English ability (Li, 2012). This is consistent with the review literature that well-prepared and effective guidance prior to the peer-feedback activities contributes to improving students’ attitudes and increasing the quality of students’ communication (Chen, 2016). This gradual feedback helps develop students’ confidence and self-esteem, the very important psychology in education.

Third, OPF should be adopted regularly in ESL/EFL study to help shaping their personalities and outlooks. As stated above, OPF is an effective way to enhance critical thinking and generate self-esteem through group collaboration, so students can unconsciously strengthen their sense of cooperation and increase their sense of achievement. This collaboration-based pedagogy is favored by Group dynamics theory (Dörnyei, 1997) and Constructive learning theory (Bada and Olusegun, 2015), stating that members in a community are complementary in the levels of intelligence, ways of thinking and even cognitive styles. Besides, group collaboration is conducive to members’ self-esteem (Bankston and Zhou, 2002). In brief, ELS/EFL learners are able to appeal to OPF to achieve comprehensive understanding and generate ideas through mutual inspiration and complement.

Fourth, OPF’s potential function is to be put into full play with recourse to abundant internet-based vehicles. To date, there has come up several learning tools based on the internet, among which are Moodle (Shang, 2022), Facebook, and blog (Chen, 2012; Wahyudin, 2018). However, these vehicles are not used as satisfactorily as anticipated in the pedagogue aspect. Therefore, what to do next is that teachers should reflect on how to choose the vehicles and associate them with different assignments or tasks in order to maximize the effects of student-centered approaches like the OPF by peers.

## Author contributions

SC and SZ collated and analyzed the data and drafted the first manuscript. TZ and YX revised the manuscript. YL and TW evaluated the theories and provided comments on the manuscript. All authors contributed to the study conception and design, edited the final version of the manuscript, and approved it for publication.

## Footnotes

1 In view of lacking an L2 writing anxiety scale for long, Cheng (2004) coined SLWAI, which was broken down into three subcomponents: cognitive (i.e., feelings that they could not understand), somatic (i.e., physical responses due to such feelings) and behavioral (i.e., avoidance because of feelings and physical effects).
