# Supplementary material for: A review of the ESL/EFL learners’ gains from online peer feedback on English writing
Source: Front Psychol. 2022 Oct 26;13:1035803. doi: 10.3389/fpsyg.2022.1035803 (PMC9645300; doi:10.3389/fpsyg.2022.1035803)
Supplement: Supplementary file 1 [file Table_1.DOCX]

**Appendix 1. Overview of the reviewed literature (chronologically)**

| Author/date | Title | Type | Level |
| --- | --- | --- | --- |
| Cai (2012) | The Contrastive Study of OPF and Online Teacher Feedback in Teaching English Writing to Chinese College Students. | E | H |
| Chang (2012) | Peer Review via Three Modes in an EFL Writing Course | E | H |
| Chen (2012) | Blog-Based Peer Reviewing in EFL Writing Classrooms for Chinese Speakers | E | H |
| Ciftci and Kocoglu (2012) | Effects of Peer E-Feedback on Turkish EFL Students’ Linguistic details | E | H |
| Ho (2012) | The Efficacy of Electronic peer feedback: From Taiwanese EFL Students’ Perspectives | E | H |
| Li (2012) | The Effects of OPF on Vocational College Learners' Acquisition of English Past Perfect Tense | E | H |
| Kitchakarn (2013) | peer feedback Through Blogs:  An Effective Tool for Improving Students’ Writing Abilities | E | H |
| Wanchid (2013) | The Use of Self-Correction, Paper-pencil peer feedback and Electronic peer feedback in the EFL Writing Class: Opportunities and Challenges | E | H |
| Yang and Meng (2013) | The Effects of Online Feedback Training on Students’ Text Revision | E | H |
| Bradley (2014) | Peer-reviewing in an intercultural wiki environment - student interaction and reflections | E | H |
| Zhang et al. (2014) | The effects of blog-mediated peer feedback on learners’ motivation, collaboration, and course satisfaction in a second language writing course | E | H |
| Chen (2016) | Technology-supported peer feedback in ESL/EFL writing classes: a research  synthesis | T |  |
| Huang (2016) | Contribution of Online Peer Review to Effectiveness of EFL Writing | E | H |
| Huang (2016) | Students and the Teacher’s Perceptions on Incorporating the Blog Task and peer feedback into EFL Writing Classes Through Blogs | E | H |
| Pham and Usaha (2016) | Blog-based peer response for L2 writing revision | E | H |
| Saeed and Ghazali (2016) | Modeling Peer Revision among EFL Learners in an Online Learning Community | E | H |
| Yang (2016) | Transforming and constructing academic knowledge through OPF in summary writing |  |  |
| Ebadi and Rahimi (2017) | Exploring the impact of online peer-editing using Google Docs on EFL learners’ academic writing skills: a mixed methods study | E | H |
| Saeed and Ghazali (2017) | Asynchronous group review of EFL writing: Interactions and text revisions | E | H |
| Cassidy and Bailey (2018) | L2 Students' Perceptions and Practices of both Giving and Receiving Online Peer-Feedback | E | H |
| Daweli (2018) | Engaging Saudi EFL Students in Online Peer Review in a Saudi University Context | E | H |
| Iksan and Halim (2018) | The Effect of E-Feedback Via Wikis on ESL Students’ L2 Writing Anxiety Level | E | H |
| Saeed et al. (2018) | A review of previous studies on ESL/EFL learners’ interactional feedback exchanges in face-to-face and computer-assisted peer review of writing | T |  |
| Saeed et al. (2018) | Engaging EFL Learners in OPF on Writing: What Does It Tell Us? | E | H |
| Wahyudin (2018) | The Impact of OPF on EFL Students’ Writing At Tertiary Level | E | H |
| Xu and Yu (2018) | An Action Research on Computer-Mediated Communication (CMpeer feedback) peer feedback in EFL Writing Context | E | H |
| Bailey and Cassidy (2019) | OPF Tasks: Training for Improved L2 Writing Proficiency, Anxiety Reduction, and Language Learning Strategies | E | H |
| Ma (2019) | Examining the role of inter-group peer online feedback on wiki writing in an EAP context | E | H |
| Pham (2020) | Computer-mediated and face-to-face peer feedback: student feedback and revision in EFL writing | E | H |
| Pham et al. (2020) | Electronic peer feedback, EFL Academic Writing and Reflective Thinking: Evidence From a Confucian Context | E | H |
| Usaha (2020) | The effectiveness of the blog-based peer response for L2 writing | E | H |
| Ghada and Nuwar (2021) | Effect of online peer review versus face-to- Face peer review on argumentative writing achievement of EFL learners | E | H |
| Elboshi (2021) | Web-Enhanced peer feedback in ESL Writing Classrooms A Literature Review | T |  |
| Putra et al. (2021) | Students’ Perceptions on OPF Practice in EFL Writing | E | H |
| Qiu and Li (2022) | A Comparative Study of the Effects of OPF and Teacher Feedback on English Writing | E | H |
| Shang (2022) | Exploring OPF and automated corrective feedback on EFL linguistic details |  |  |
| Sun and Zhang (2022) | Effects of Translanguaging in OPF on Chinese University English-as- a-Foreign-Language Students’ Second Language Linguistic details | E | H |

**T = theoretical, E = empirical, H = higher education.*
